# Comment on: “Health outcomes and female genital mutilation/cutting: how much is due to the cutting itself?”

**DOI:** 10.1038/s41443-023-00667-8

**Published:** 2023-02-09

**Authors:** Birgitta Essén

**Affiliations:** Department of Women’s and Children’s Health/IMHm, WHO Collaborating Centre for Migration and Health Data, Uppsala University, Uppsala, Sweden

**Keywords:** Diagnosis, Reproductive signs and symptoms

Johnson-Agbakwu et al. [1] raise a striking hypothesis: that in certain populations of migrant women affected by female genital mutilation/cutting (FGM/C), it may not be the FGM/C—per se—but rather, experiences of discrimination, that “drive” various FGM/C-associated physical and mental health problems. This hypothesis stands in contrast to the view generally adopted by the World Health Organization (WHO), which implies a clear cause and effect relationship between FGM/C as such (i.e., the “common denominator” for all “cut” women, irrespective of their particular circumstances or background) and a wide range of adverse health outcomes [2]. The study by Johnson-Agbakwu et al. thus challenges a common assumption: namely, that a direct causal relationship can reliably be drawn between a genital wound typically incurred during childhood (which subsequently heals) and specific psychological and reproductive health complications reported, often many years later, in adulthood.

The authors have good reasons for questioning this assumption. Despite countless scientific papers claiming statistical associations—and implying causation—between FGM/C status and adverse health outcomes, these associations are rarely supported by biologically plausible explanations [3, 4], with a few notable exceptions [5] (e.g., being cut under unhygienic circumstances is known to be causally associated with immediate acute risks of hemorrhage, infection, and tissue swelling with urine retention). For example, it is often assumed that scarring from FGM/C—whether Type 1, 2 or 3—is causally responsible for prolonged and obstructed labor. However, anatomically this is implausible because prolonged labor is caused by dysfunction of the uterine muscle: obstructed labor is explained by a mechanical obstruction due to disproportionality in size between the fetus and the birth canal, rather than to scar tissue outside the birth canal. Indeed, no consensus has been reached by WHO and scholars as to causality or explanatory mechanism [4, 6]. Thus, questioning an assumption of cause and effect between FGM/C and certain long-term health difficulties is of considerable scientific value and should, in my view, be seen as the main contribution of the authors’ study.

Speaking generally, we have very little idea of the specific mechanisms that might link childhood experiences of FGM/C to health outcomes later in life. If we consider the results of existing systematic reviews and meta-analyses, we can begin to see why. Firstly, with respect to some forms of FGM/C, such as “nicking” (or other forms that might be categorized within WHO Type 4 or 1a), there are essentially no data available with which one could even begin to assess a relationship between childhood exposure to the procedure and any long-term outcomes [5, 7]. Secondly, systematic reviews of other forms of FGM/C reveal an overall high frequency of poor quality data coming from studies of low methodological rigor [8–10], while meta-analyses that have pooled data with the ambition to present more stable results than can be derived from any single study confirm that “the quality of evidence for all outcomes as being too low to warrant conclusions about a causal relationship between FGM/C and obstetric complications” [11]. Indeed, the majority of published studies on FGM/C have serious limitations and flaws irrespective of design, whether one considers case series, cross sectional, or cohort studies. These flaws include unreliable measurements, non-validated questionnaires, incommensurate (i.e., between studies) materials, unrepresentative samples of girls and women, missing anatomical descriptions, low response rates, improperly collected data, misclassification, recall bias, selective reporting and other reporting bias, and lack of reproducibility in other settings [3, 8, 12, 13].

Against this backdrop, let us now consider the paper by Johnson-Agbakwu et al. The authors’ study is entitled: “Health Outcomes and Female Genital Mutilation/Cutting: How Much is Due to the Cutting Itself?” Given the phrase “due to” in the title, one might expect a classic study design assessing independent (i.e., FGM/C) and dependent (i.e., health outcomes) variables in terms of the aforementioned hypothetical relation of cause and effect. However, the study design they actually pursue seems to be oriented differently: i.e., to determine whether self-reported discrimination among Somali migrants living in the United States predicts various adverse health outcomes, independently of FGM/C status.

The intention was to measure clinically significant psychological distress and self-reported FGM/C-related health morbidity, examined against self-reported experiences of everyday discrimination. For psychological distress, the authors used a questionnaire with a validated scale for measuring refugee mental health disorders, the Refugee Health Screener 13 (RHS-13) [14]. FGM/C-related health morbidity was, as they state, “analyzed dichotomously based on whether the participant had experienced any of 28 gynecological, sexual, or obstetric health concerns in the past 2 years. Examples include difficulty passing urine, recurrent urinary tract or genital infections, pain with intercourse, difficulty getting pregnant, emergency C-section, and postpartum hemorrhage” [1]. Finally, to assess everyday experiences of discrimination, participants were asked to report the frequency with which they recalled having had certain experiences, as follows: “treated with less courtesy/respect than other people; poorer service than others at restaurant/store; people act afraid of you; threatened/harassed; people act as if they think you are not smart” (10.1017/S1742058X11000087). A five item mean index was calculated, based on scores ranging from 1 = never experienced to 5 = experienced at least once a week (range from 1–5, *α* = 0.77).

Surprisingly, the authors did not ask any questions related to perceived discrimination or other activities occurring during “healthcare encounters”. That is, none of the items shown to participants were specific to experiences with health service providers or medical institutions; rather, they only concerned general self-perceived events from everyday life, or events taking place in other contexts (e.g., restaurants/stores). With the items they used, the authors found a significant correlation between perceived everyday discrimination and negative health outcomes; but the nature of this relationship—in terms of a potential mechanism—is not explored. How does receiving poor service when visiting a restaurant or store, for example, relate to difficulties in getting pregnant or the occurrence of a genital tract infection? How does my sense that others are acting as though they think I am not smart relate to the fact that I experienced postpartum hemorrhage during my last delivery? How does the perception that others are afraid of me relate to my difficulty in passing urine? And so on.

Theoretically, perceptions of poor treatment in healthcare encounters might lead to a change in care-seeking behavior, which in turn may lead to risk for suboptimal care due to a patient’s avoidance/delay. But in order to draw those conclusions, a more precisely developed theory and stronger empirical data from well-designed studies would be needed. It is possible that those in the present study who reported more instances of perceived discrimination in general or in other contexts would, if asked, also have reported more instances of perceived discrimination in healthcare contexts specifically, but that is only a speculation. And even if so, there would still need to be more direct evidence of a mechanism linking those data to specific adverse outcomes among women with (or without) FGM/C before it would be appropriate to infer the suggested relationship of discrimination driving the adverse outcomes.

Over the years, several researchers have tried to draw attention to the fact that other explanations must be sought for poor health outcomes in women affected by FGM/C than simply the presence of scar tissue or other anatomical effects of a childhood injury to the vulva [15–18]. The study by Johnson-Agbawaku et al. contributes to this much-needed expansion of scientific inquiry. However, one cannot draw causal conclusions from their data. In the abstract to their paper, Johnson-Agbakwu et al. hedge their language, stating that their findings are only “consistent with” views according to which “discrimination drives negative outcomes.” But in the lengthy discussion section following the reporting of their correlational results, they refer to the “likelihood” that “social factors such as discrimination and support may play a larger role in health than FGM/C” and otherwise strongly imply a causal relationship.

To fully understand the inequity in reproductive health outcomes among women of the Somali diaspora that has been known for decades [15, 17, 19], clinicians and researchers must, as the authors forcefully argue, think outside the box and resist a reductive, overly-narrow focus on these women’s genitalia. Proximate explanations including care-seeking behavior, comfort or ability in navigating a new healthcare system, sources of support or discouragement, miscommunications due to language or cultural barriers causing treatment delays, and other hypothesized factors including, as the authors propose and explore, perceptions of everyday discrimination, all must be carefully investigated. But in all cases, just as we must not reflexively attribute specific health problems to FGM/C, so too must we take care to avoid going beyond our data with respect to proposed psychosocial explanations.
